# A pragmatic multi-centre randomised controlled trial of fluid loading and level of dependency in high-risk surgical patients undergoing major elective surgery: trial protocol

**DOI:** 10.1186/1745-6215-11-41

**Published:** 2010-04-16

**Authors:** Brian H Cuthbertson, Marion K Campbell, Stephen A Stott, Luke Vale, John Norrie, John Kinsella, Jonathan Cook, Julie Brittenden, Adrian Grant, FOCCUS Study Group

**Affiliations:** 1 Department of Critical Care Medicine, Sunnybrook Health Sciences Centre, Toronto, Ontario, Canada; 2 Health Services Research Unit, Health Sciences Building, University of Aberdeen, Foresterhill, Aberdeen, UK; 3 Intensive Care Unit, Aberdeen Royal infirmary, Westburn Road, Aberdeen, UK; 4 Robertson Centre for Biostatistics, University of Glasgow, Glasgow, UK; 5 Department of Anaesthesia Pain and Critical Care, University of Glasgow, Glasgow, UK; 6 Department of Surgery, University of Aberdeen, Foresterhill, Aberdeen, UK; 7 Institute of Applied Health Sciences, Health Sciences Building, University of Aberdeen, Foresterhill, Aberdeen, UK; 8 Health Economics Research Unit, Polwarth Building, University of Aberdeen, Foresterhill, Aberdeen, UK

## Abstract

**Background:**

Patients undergoing major elective or urgent surgery are at high risk of death or significant morbidity. Measures to reduce this morbidity and mortality include pre-operative optimisation and use of higher levels of dependency care after surgery. We propose a pragmatic multi-centre randomised controlled trial of level of dependency and pre-operative fluid therapy in high-risk surgical patients undergoing major elective surgery.

**Methods/Design:**

A multi-centre randomised controlled trial with a 2 * 2 factorial design. The first randomisation is to pre-operative fluid therapy or standard regimen and the second randomisation is to routine intensive care versus high dependency care during the early post-operative period. We intend to recruit 204 patients undergoing major elective and urgent abdominal and thoraco-abdominal surgery who fulfil high-risk surgical criteria. The primary outcome for the comparison of level of care is cost-effectiveness at six months and for the comparison of fluid optimisation is the number of hospital days after surgery.

**Discussion:**

We believe that the results of this study will be invaluable in determining the future care and clinical resource utilisation for this group of patients and thus will have a major impact on clinical practice.

**Trial Registration:**

Trial registration number - ISRCTN32188676

## Background

Patients undergoing major elective or urgent surgery are at high risk of death or significant morbidity. Death around the time of surgery accounts for nearly one in 20 of all deaths in the UK and the rates are not significantly declining. Amongst the patients who survive, many suffer major complications with subsequent impairment of health and quality of life.

The National Confidential Enquiry into Peri-operative Deaths shows that such deaths are commonly associated with cardiac complications [1,2]. Putative contributory factors include a lack of High Dependency Unit (HDU) and Intensive Care Unit (ICU) beds, and suboptimal pre- and post-operative ward care. The Department of Health's report "Comprehensive Critical Care" [3] proposed that an HDU facility should care for frail patients who require monitoring or specialised analgesia after surgery without the immediate availability of medical staff, whereas an ICU-based HDU facility should care for patients requiring more detailed observation or intervention, including immediate availability of experienced medical staff. This simple system offers much to be commended as an attempt to allow more cost-effective and flexible utilisation of critical care services. However, there has been little research on the effects of ICU care alone on outcome from major surgery and these guidelines are based on expert opinion rather than empirical evidence. Further, they have not been routinely adopted and implemented into current clinical practice; only around 60% of hospitals have ward-based surgical HDU facilities and fewer hospitals have ICU-based HDU facilities [1,2]. "Comprehensive Critical Care" [4] also identified a need for a greater evidence-base to justify the huge clinical resources that are currently invested in critical care services throughout the UK. The question remains whether routine ICU-based postoperative care improves outcomes after major surgery in a cost-effective manner.

There is also evidence that peri-operative risk can be reduced by specific interventions applied before surgery. Pre-operative optimisation and supranormalisation aims to 'optimise' cardiac index and oxygen delivery through a pre-operative fluid and inotrope strategy that is maintained in the intra-operative and early post-operative periods [5-7]. Early randomised studies suggested a significant outcome advantage from this strategy. Wilson *et al *[6] found a mortality of 17% in the control group compared to 3% in the treatment group and claimed that this represented a cost-effective improvement in peri-operative care [6]. This study has, however, been criticised because of the poor outcome in the control group. Amongst those who consider pre-operative optimisation and supranormalisation to be effective, there is debate as to which aspect of the strategy brings about the reduction in mortality. Furthermore, the practicality of 'full' pre-operative optimisation and supranormalisation is limited because ICU-based care is required both before and after surgery. This has led to consideration of whether components that could be applied outside an ICU setting might still be useful.

This proposal is for a randomised controlled trial to test two of the most promising (but controversial) aspects of peri-operative care: firstly, a simple and practical approach to pre-operative preparation using ward-based intravenous fluid therapy; and secondly, the routine provision of intensive care facilities after surgery. We aimed to test whether ward-based pre-operative fluid loading and post-operative higher levels of dependency are effective and cost-effective in high-risk surgical patients undergoing major elective and urgent surgery.

## Methods/Design

A multi-centre, prospective, randomised, controlled trial with a partial 2 * 2 factorial design conducted in three UK hospitals, coordinated from the Centre for Healthcare Randomised Trials (CHaRT) in the Health Services Research Unit, University of Aberdeen [8,9]. The design features and estimates used in this protocol were further informed by the results of a 15-week pilot of the proposed protocol undertaken in Aberdeen Royal Infirmary, during which 23 patients were recruited.

### Research question

This trial aims to address the question whether: 1) ward-based pre-operative fluid optimisation and/or 2) routine post-operative ICU-based care for high risk surgical patients undergoing major elective and urgent surgery improves outcome and is cost-effective.

### Interventions to be evaluated

Patients will be randomised to a) pre-operative or standard regimen; and then secondly to b) ICU or HDU for post-operative care. The partial nature of the factorial design recognises that some patients may not be able to be randomised to the ICU or HDU comparison (as ICU beds cannot always be guaranteed).

### Pre-operative fluid therapy or standard regimen

In the pre-operative fluid therapy group patients will be electively commenced on pre-operative fluid therapy (25 ml/kg) using Hartmann's solution over six hours before surgery in the ward setting. In the standard fluid regimen no routine pre-operative fluid therapy will be given. All patients receiving bowel preparation will be given an additional 10 ml/kg Hartmann's solution in the 12 hours before surgery irrespective of trial group allocation (as this is deemed to be best clinical practice). Patients receiving "Klean-prep" bowel preparation with 4 litres of oral fluid will not receive additional IV fluids.

### ICU or HDU for post-operative care

The ICU care group will have initial post-operative care undertaken in the ICU under the care of ICU clinicians [3]. The HDU care group will have initial post-operative care undertaken in a surgical HDU under the care of the surgical team [3].

All non-protocol fluid prescriptions and other management decisions (including the movement of the patients through differing levels of dependency) will be made by the clinically responsible medical staff.

### Inclusion criteria

Inclusion criteria include patients undergoing major elective and urgent abdominal and thoraco-abdominal surgery who fulfil high-risk surgical criteria [10] who have signed informed consent. These high risk criteria include high risk type of surgery, presence of ischaemic heart disease, history of congestive heart failure, history of cerebrovascular disease, insulin therapy for diabetes and pre-operative serum creatinine > 2 mg/dl. Patients who undergo open, laparoscopic or laparoscopically-assisted surgery will be eligible.

### Exclusion criteria

Exclusion criteria will include New York Heart Association grade IV heart failure; clinician concern about safety of interventions; emergency surgery; chronic renal failure/creatinine > 300 umol.L^-1^, lack of informed consent; age < 16 years; pregnancy; major hepatic surgery, expected survival < 6 months.

### Study participants

Participants will be recruited from the elective operating schedules in the recruiting hospitals and informed consent obtained. Patients will, in general, be identified following a pre-assessment visit at a local hospital or following their initial appointment with the surgeon. They will be sent an information leaflet prior to their hospital admission, or given one at their appointment. Informed consent will be sought from these patients on their admission to hospital. Participants will be randomised through an interactive voice response (IVR) automated telephone randomisation service. A minimisation algorithm will be used, incorporating centre, age, sex and type of surgery [11]. All participants will be followed up daily for one week for major morbidity and mortality, then at hospital discharge and then one, three and six months after surgery for survival and quality of life.

### Outcome measure

#### a) Pre-operative fluid therapy or standard regimen

The primary outcomes outcome is the number of hospital days after surgery (length of stay).

#### b) Routine ICU or HDU for post-operative care [3]

The primary outcome is cost-effectiveness at six months, measured by the Net Benefit statistic which is calculated using the following equation: ((λ * QALY) - costs) where λ indicates society's 'willingness to pay' (λ is typically set at £20,000 and this value will be used for the main analysis); QALYs are calculated using EQ-5D scores and costs include both primary and secondary care costs.

For both comparisons, secondary outcomes include measures of health status at one month after surgery measured using SF-36 by patient completed survey [12,13](with appropriate adjustment to accommodate for patients who die); changes in health status and quality of life over 6 months after surgery and quality of life at 48 hours, three and six months after surgery measured using SF-36 [12,13] and EQ-5D [14]; QALY calculated using EQ-5D. Other secondary measures include measures of health care costs including full hospital costs (driven by critical care utilisation and hospital length of stay) and primary care costs. Mortality will be measured using time-to-event analysis and the level of major morbidities in hospital using Post-Operative Morbidity Survey (POMS) [15]. The decision to discharge the patient from hospital was made by the caring team with no involvement of the study personnel. Study outcome measured during hospital stay were measured by study personnel not blinded to the intervention. Outcomes measured after hospital discharge were measured by study personnel blinded to the intervention.

### Sample size

As the smallest number of patients will be available for the comparison of ICU and HDU care (as not all patients will be able to be randomised to this comparison), this comparison drives the sample size calculation. We aim to be able to detect 0.45 standard deviations of a difference in the primary outcome (mean Net Benefit Statistic) between the two groups. For example, if the standard deviation of Net Benefits in both groups was about £10,000, this would equate to being able to detect a difference in the mean Net Benefit of £4,500. To be able to detect this level of difference with 80% power and a 5% level of significance, we will recruit 156 patients to this comparison. We further estimate that around 10% of patients will not have data available on all components of the cost-effectiveness at six months (which include data which is self-reported) and as such have inflated the number required to be recruited to this comparison to 174.

In the pilot study we found a 10% failure rate to progress from the first randomisation (pre-operative fluid) to the second randomisation (ICU versus HDU) because ICU care was not available. As such, the total sample size required to be recruited to the comparison of pre-operative fluid therapy or standard management needs to be at least 10% greater than that being sought for the ICU/HDU comparison. Conservatively, we have assumed that 15% will not be able to be randomised to the ICU/HDU randomisation and thus estimate that 204 patients be recruited to the study in total.

**Figure 1:**
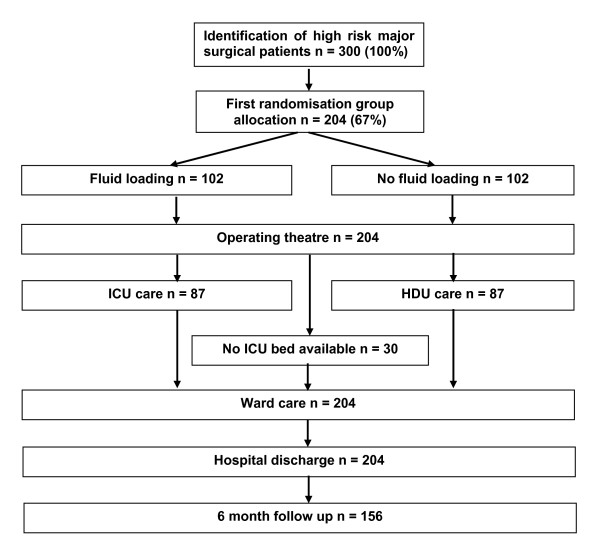
**Trial patient pathway**.

We have no reason to expect any loss to follow up for the primary outcome of the pre-operative fluid comparison (as the primary outcome is number of days in hospital after surgery). Ideally this comparison would have sufficient power to detect a 0.5 standard deviation change in number of days in hospital, with 80% power and 5% significance (requiring data on 128 patients to be available for analysis). In fact, a total sample size of 204 patients would allow a difference of 0.4 standard deviations of a difference to be detected with at least 80% power at a 5% level of significance (and over 90% power to detect a 0.5 standard deviation difference). The trial patient pathway is presented in figure 1.

The study would also have approximately 90% power to address the health related quality of life secondary outcomes. Previous quality of life studies suggest a difference in means of 0.5 of a standard deviation in Health Related Quality of Life scores reflects a clinically important change. The pilot study indicated that such a difference would translate to around five points in the mean physical component score of the SF-36 questionnaire.

### Trial management

The pilot study suggested that non-compliance with the proposed protocol will be uncommon. Nevertheless, we recognise that ICUs must have the ability to discharge trial patients early according to clinical need; this will be monitored closely and quality assurance monitoring performed. Weekly progress meetings will be held in each clinical centre and at the study Data Centre at CHaRT. The applicants will form a Trial Management Group, which will meet monthly. CHaRT will also perform detailed and regular site reviews and visits to ensure recruitment. If they are not achieved then they will undertake further actions to remedy this as required. The trial will be supervised by a Trial Steering Committee with an independent Chair. This Committee will oversee the trial and monitor progress against the milestones. An independent Data Monitoring Committee (DMC) will, during the period of recruitment to the trial, be provided with interim analyses, in strict confidence, together with any other analyses that the committee may request. The frequency of any interim analyses will be based on the judgement of this Committee.

### Data analysis

The statistical analyses will be based on all people randomised who are retained in the study until the primary outcome time points, irrespective of subsequent compliance to treatment allocation. The principal comparisons will be a) all patients randomised to fluid optimisation versus all patients not allocated fluid optimisation and b) all patients randomised to routine ICU care versus all patients randomised to HDU for post-operative care.

Trial analysis will be undertaken using standard methods for two-group comparisons for continuous, binary and time-to-event outcomes [9]. All statistical analyses will be pre-specified in a Statistical Analysis Plan which will be agreed before any unblinded data is seen. Length of stay will be analysed using linear regression adjusted for the minimisation variables [11]. For the net benefit statistic, linear regression will also be used with adjustment for minimisation variables and the baseline EQ-5D score. Bootstrapping of the confidence interval for the mean difference in net-benefit will be performed and statistical significance determined on the basic of this interval including zero. Further details on the generation of the components of the net-benefit statistic are given in the economic analysis section. Health related quality of life measure will be analysed similarly. Mortality will be analysed using a Cox-proportional hazards model adjusted for minimisation factors. POMS will be analysed using logistic regression adjusted for minimisation covariates. A two-sided 5% significance level will be applied as evidence of statistical significance for the analyses. Corresponding confidence intervals will be presented.

For the fluid randomisation we plan to include patients recruited into the pilot study within the analysis as they have undergone the same interventions and have the same outcome measures collected (hospital length of stay). Pilot study data will not be used in the analysis of ICU length of stay as the primary outcome time point was changed from 1 year to 6 months as a result of the pilot.

A single principal analysis is planned six months after the last person is recruited. If considered appropriate, follow-up of recruits will be extended at this time and a corresponding latter analysis will also be conducted once the latter follow-up data is available. Secondary subgroup analyses will be investigated through tests for interaction and will include patients with co-morbidities, cardiac risk [11], urgency and type of surgery. Stricter levels of statistical significance (2-sided 1% significance level) will be applied reflecting the exploratory nature of these subgroup analyses.

### Economic analysis

Based on experience gained through the pilot study, costs to the NHS will be elicited using a "top-down" method. Resource data will be collected on hospital costs by patient follow up, case note review and through the Patient Admissions System. The main drivers of hospital costs will be critical care utilisation and hospital length of stay. Primary care resource utilisation, patient costs and costs to carers will be identified by telephone interview. NHS resource usage will be costed using appropriate unit costs. Incremental cost per quality adjusted life year will be calculated using data elicited from the EQ-5D questionnaire. For patients who die, a quality of life score of zero will be applied for measurements after death [14,16,17]. Uncertainty will be explored using both stochastic (which will utilise the net benefits data) and other forms of sensitivity analysis [17]. The stochastic analysis will explore the impact of using different values for society's willingness to pay for a QALY (λ) and these data will be presented in the form of a cost-effectiveness acceptability curve. Sensitivity analysis will also be used to explore the impact of imputing any missing data.

### Ethics approval

The Multicentre Research Ethics Committee for Scotland has approved the study for the UK (Ref 04/MRE10/76).) The trial has been registered in a public trials registry (registry number ISRCTN32188676). The trial will be conducted according to the principles of good practice provided by Research Governance Guidelines and the Data Protection Act 1998. The trial is sponsored by the University of Aberdeen. The Trial Management Group, through the Trial Steering Committee, will ensure that adequate systems are in place for monitoring the quality of the study (compliance with Good Clinical Practice (GCP)) and appropriate expedited (when appropriate) and routine reports of adverse effects.

## Discussion

It is clear from the existing evidence based that there is a lack of evidence to guide clinicians and health service managers on the optimal peri-operative management of high risk patients. This group of patients has very significant morbidity and mortality and strategies need to be identified to improve these outcomes. We are conducting this randomised controlled trial to attempt to answer two important research questions in this patient group which will supply highly useful evidence on the optimal management in this group and help guide clinicians as well as health service managers in their prioritisation and decision making.

## Competing interests

The authors declare that they have no competing interests.

## Authors' contributions

BHC has participated fully in the design of this study and in the writing this paper and has seen and approved the final version of the paper. MKC has participated fully in the design of this study and in the writing this paper and has seen and approved the final version of the paper. SAS has participated fully in the design of this study and in the writing this paper and has seen and approved the final version of the paper. LV has participated fully in the design of this study and in the writing this paper and has seen and approved the final version of the paper. JN has participated fully in the design of this study and in the writing this paper and has seen and approved the final version of the paper. JK has participated fully in the design of this study and in the writing this paper and has seen and approved the final version of the paper. JC has participated fully in the design of this study and in the writing this paper and has seen and approved the final version of the paper. JB has participated fully in the design of this study and in the writing this paper and has seen and approved the final version of the paper. AG has participated fully in the design of this study and in the writing this paper and has seen and approved the final version of the paper.

## Author's information

BHC is an intensive care clinician and clinical researcher and trialist at the Department of Critical Care Medicine, Sunnybrook Health Sciences Centre, Toronto, Ontario, Canada and holds the status of honorary Professor at the University of Aberdeen. MKC is a clinical trialist and Director of the Health Services Research Unit at the University of Aberdeen, Aberdeen, UK. SAS is an intensive care clinician in the Intensive Care Unit, Aberdeen Royal Infirmary, Aberdeen, UK. JK is an intensive care clinician and clinical researcher at the University of Glasgow and Intensive Care Unit in Glasgow Royal Infirmary. JN is a Professor of Medical Statistics at the Robertson Centre for Biostatistics at the University of Glasgow, UK. JC is a medical statistician in the Health Services Research Unit at the University of Aberdeen, Aberdeen, UK. LV is a Professor of Health Technology Assessment in the Health Services Research Unit and Health Economics Research Unit at the University of Aberdeen, Aberdeen, UK. JB is a reader in vascular surgery and consultant vascular surgeon at the University of Aberdeen and Vascular Surgery Department, Aberdeen Royal Infirmary, Aberdeen, UK. AG is a Professor of Health Services Research and clinical trialist in the Institute of Applied Health Sciences at the University of Aberdeen, Aberdeen, UK.

## References

[B1] NCEPOD Annual report 1999-2002 2003 NCEPOD London http://www.ncepod.org.uk/

[B2] Scottish Audit of Surgical Mortality Annual report 1996 1997 SASM; Scotland http://www.sasm.org.uk/

[B3] Scottish Executive; Health Department "Better critical care" 2000 Edinburgh: Scottish Executive http://www.sehd.scot.nhs.uk/publications/report.PDF

[B4] Department of Health Comprehensive Critical Care. A review of Adult critical care services. London 2000 http://www.dh.gov.uk/en/Publicationsandstatistics/Publications/PublicationsPolicyAndGuidance/DH_4006585

[B5] Shoemaker WC Appel PL Kram HB Waxman K Lee T-S Prospective trial of supranormal values of survivors as therapeutic goals in high-risk surgical patients Chest 1988 94 1176 1186 10.1378/chest.94.6.1176 3191758

[B6] Wilson J Woods I Fawcett J Whall R Dibb W Morris C McManus E Reducing the risk of major elective surgery: randomised controlled trial of pre-operative optimisation of oxygen delivery BMJ 1999 318 1099 1103 10213716 10.1136/bmj.318.7191.1099 PMC27840

[B7] Kern JW Shoemaker WC Meta-analysis of hemodynamic optimization in high-risk patients Critical Care Medicine 2002 30 1686 1692 10.1097/00003246-200208000-00002 12163777

[B8] Medical Research Council A framework for development and evaluation of RCTs for complex interventions to improve health 2000 http://www.mrc.ac.uk/pdf-mrc_cpr.pdf

[B9] McAlister FA Straus SE Sackett DL Altman DG Analysis and reporting of factorial trials: a systematic review JAMA 2003 2899 2545 53 10.1001/jama.289.19.2545 12759326

[B10] Lee TH Marcantanio ER Mangione CM Derivation and prospective validation of a simple index for prediction of cardiac risk of major non-cardiac surgery Circulation 1999 100 1043 49 10477528 10.1161/01.cir.100.10.1043

[B11] Scott NW McPherson GC Ramsay CR Campbell MK The method of minimization for allocation to clinical trials: a review Control Clin Trials 2002 23 662 74 10.1016/S0197-2456(02)00242-8 12505244

[B12] Hayes JA Black NA Jenkinson C Young JD Rowan KM Daly K Ridley S Outcome measures for adult critical care: a systematic review Health Technology Assessment 2000 4 1 111 11074394

[B13] Garratt AM Ruta DA Abdalla MI Russell IT SF36 health survey questionnaire: II Responsiveness to changes in health status in four common clinical conditions Quality in Health Care 1994 3 186 192 10.1136/qshc.3.4.186 10140232 PMC1055239

[B14] Dolan P Gudex C Kind P Williams A A social tariff for EuroQol: Results from a UK General Population Survey Centre for Health Economics Discussion Paper 138. University of York 2002

[B15] Bennett-Guerrero E Welsby I Dunn TJ Young LR Wahl TA Diers TL Phillips-Bute BG Newman MF Mythen MG The use of a postoperative morbidity survey to evaluate patients with prolonged hospitalization after routine, moderate-risk, elective surgery Anesth Analg 1999 89 514 519 10.1097/00000539-199908000-00050 10439777

[B16] Briggs A Gray A Handling uncertainty when performing economic evaluation of healthcare interventions Health Technology Assessment 1999 3 2 10448202

[B17] Carli F Mayo N Klubien K Schricker T Trudel J Belliveau P Epidural analgesia enhances functional exercise capacity and health-related quality of life after colonic surgery: results of a randomized trial Anesthesiology 2002 97 540 9 10.1097/00000542-200209000-00005 12218518

